# Fragmentary Proving of Potassium Chlorate

**Published:** 1892-12

**Authors:** E. Rushmore

**Affiliations:** Plainfield, N. J.


### INTERNATIONAL HAHNEMANNIAN ASSOCIATION MEETING OF 1892.

### FRAGMENTARY PROVING OF POTASSIUM CHLORATE.

E. Rushmore, M. D., Plainfield, N. J.

Miss ——, brunette, short, æt. about fifty, had goitre in early life, which disappeared under the application of Iodine. Some time afterward a fibroid tumor of the womb appeared, subperitoneal as diagnosticated many years ago. It still remains. Under the advice of friends, non-medical, she was induced to take the Potassium Chlorate, crude, dissolved in water in daily doses. When coming to me lately for treatment she reported the following as the effects of the drug she had taken :

Increased moral irritability. Felt dreadfully dull and stupid.

Dizzy on stooping and rising.

Slight headache over the eyes.

Objects appeared double, beside each other.

Face swelled so she could hardly see on rising in the morning.

Smarting of the tongue. It took away her desire for acids.

Diminished appetite.

Much commotion and flatulence in the abdomen.

Increased urine.

Dreams worse, terrible, of things never thought of.

General coolness ; shivering on cold days ; seemed as if it cooled off her blood.

Clumsiness. General bloated feeling.

